# Influenza Vaccine Effectiveness in Preventing Influenza A(H3N2)-Related Hospitalizations in Adults Targeted for Vaccination by Type of Vaccine: A Hospital-Based Test-Negative Study, 2011–2012 A(H3N2) Predominant Influenza Season, Valencia, Spain

**DOI:** 10.1371/journal.pone.0112294

**Published:** 2014-11-13

**Authors:** Joan Puig-Barberà, Juan García-de-Lomas, Javier Díez-Domingo, Alberto Arnedo-Pena, Montserrat Ruiz-García, Ramón Limón-Ramírez, Silvia Pérez-Vilar, José Luis Micó-Esparza, Miguel Tortajada-Girbés, Concha Carratalá-Munuera, Rosa Larrea-González, Juan Manuel Beltrán-Garrido, Maria del Carmen Otero-Reigada, Joan Mollar-Maseres, Patricia Correcher-Medina, Germán Schwarz-Chavarri, Vicente Gil-Guillén, for the Valencia Hospital Network for the Study of Influenza and Respiratory Virus Disease

**Affiliations:** 1 Fundación para el Fomento de la Investigación Sanitaria y Biomédica de la Comunitat Valenciana (FISABIO), Valencia, Spain; 2 Centro de Salud Pública de Castellón, Castellón, Spain; 3 Instituto Valenciano de Microbiología, Valencia, Spain; 4 Hospital de la Plana, Vila-real, Spain; 5 Hospital Aranau de Vilanova, Valencia, Spain; 6 Hospital Doctor Peset, Valencia, Spain; 7 Hospital San Juan, Alicante, Spain; 8 Cátedra de Medicina de Familia, Departamento de Medicina Clínica, Universidad Miguel Hernández, San Juan, Alicante, Spain; 9 Hospital General de Castellón, Castellón, Spain; 10 Hospital Universitario y Politécnico La Fe, Valencia, Spain; 11 Hospital Lluis Alcanyis, Xativa, Spain, **12** Centro de Salud San Blas, Alicante, Spain, **13** Hospital de Elda, Elda, Spain; 12 Centro de Salud San Blas, Alicante, Spain; 13 Hospital de Elda, Elda, Spain; University of Calgary & ProvLab Alberta, Canada

## Abstract

**Background:**

Most evidence of the effectiveness of influenza vaccines comes from studies conducted in primary care, but less is known about their effectiveness in preventing serious complications. Here, we examined the influenza vaccine effectiveness (IVE) against hospitalization with PCR-confirmed influenza in the predominant A(H3N2) 2011–2012 influenza season.

**Methods:**

A hospital-based, test-negative study was conducted in nine hospitals in Valencia, Spain. All emergency admissions with a predefined subset of symptoms were eligible. We enrolled consenting adults age 18 and over, targeted for influenza vaccination because of comorbidity, with symptoms of influenza-like-illness within seven days of admission. We estimated IVE as (1-adjusted vaccination odds ratio)*100 after accounting for major confounders, calendar time and recruitment hospital.

**Results:**

The subjects included 544 positive for influenza A(H3N2) and 1,370 negative for influenza admissions. Age was an IVE modifying factor. Regardless of vaccine administration, IVE was 72% (38 to 88%) in subjects aged under 65 and 21% (−5% to 40%) in subjects aged 65 and over. By type of vaccine, the IVE of classical intramuscular split-influenza vaccine, used in subjects 18 to 64, was 68% (12% to 88%). The IVE for intradermal and virosomal influenza vaccines, used in subjects aged 65 and over, was 39% (11% to 58%) and 16% (−39% to 49%), respectively.

**Conclusions:**

The split-influenza vaccine was effective in preventing influenza-associated hospitalizations in adults aged under 65. The intradermal vaccine was moderately effective in those aged 65 and over.

## Introduction

Influenza runs on temperate zones as yearly seasonal epidemics [1]. These epidemics are associated with excess morbidity, hospitalizations and deaths [2]–[4]. Vaccination is considered the most effective strategy for preventing influenza and is recommended for individuals at high risk of serious complications [5].

Influenza vaccine effectiveness (IVE) varies seasonally depending on the antigenic proximity between the vaccine and circulating strains due to evolutionary drift [6]–[8] or altered antigenicity of the egg-derived strains in the vaccine [9]–[12]. IVE against laboratory-confirmed influenza in adults aged under 65 years old is described as moderate, whereas the evidence of protection in adults aged over 65 has been questioned [8]. Most evidence of the effectiveness of influenza vaccines comes from studies conducted in primary care, however, studies of the effectiveness in preventing serious complications, defined as admissions with influenza, are needed [13], as limited data on the effectiveness of the vaccines in preventing hospitalizations restrains advocacy for vaccination and the accuracy of estimates of the cost effectiveness of offering influenza vaccines [14].

The annual assessment of IVE against specific influenza-related outcomes is possible using real-time reverse transcription-polymerase chain reaction (rtRT-PCR) to ascertain influenza infection [15] and comparing the odds of being vaccinated among positive compared to negative for influenza admissions [16]–[20]. IVE estimates obtained from this test-negative approach are considered minimally biased, given that certain assumptions and conditions are met [20]–[22]. The test-negative study design has been used with consistent results [8].

The fact that for the 2011–2012 season three types of vaccine were offered free of charge to all inhabitants in Valencia (Spain) belonging to target groups for influenza vaccination [5], [23], and the existence of the Valencia Hospital Network for the Study of Influenza and Respiratory Virus Disease (VAHNSI) [18], gave us the opportunity to estimate the IVE in preventing confirmed influenza hospitalizations by age group and type of vaccine.

## Methods

### Ethics Statement

The Ethics Research Committee of the Centro Superior de Estudios en Salud Pública (CSISP) approved the protocol of the study. All participants gave written informed consent before inclusion.

### Study settings

We performed a prospective test-negative study. The study was conducted in nine health districts in Valencia, Spain. The hospitals in the participating districts, representing for the Valencia Hospital Network for the Study of Influenza and other Respiratory Virus Diseases (VAHNSI), provided health care services to 1,783,472 inhabitants, aged 18 years and over.

We considered that the influenza season began when two or more positive influenza hospitalizations were identified in two consecutive weeks and the season was considered to have ended in the week in which no identifications were observed for at least two consecutive weeks.

Three vaccines were acquired by public tender by the regional government and centrally distributed to be offered free of charge to groups targeted for influenza vaccination [23]: a split trivalent classical intramuscular vaccine (Gripavac, Sanofi Pasteur MSD, Lyon, France), a virosomal trivalent subunit vaccine (Inflexal-V, Crucell, Leiden, The Netherlands), and a split trivalent intradermal vaccine (Intanza 15 micrograms, Sanofi Pasteur MSD, Lyon, France). The split trivalent intramuscular (IM) influenza vaccine was offered to individuals belonging to groups targeted for influenza vaccination aged under 65 in all health districts. The virosomal vaccine was exclusively distributed in four health districts out of nine participating in the study and the intradermal vaccine was exclusively distributed in the other five districts. Both were aimed at individuals aged 65 or older.

From Monday to Saturday, study staff screened all previous 48-hour emergency admissions to identify eligible subjects because their indication for hospitalization could be related to influenza (Table S1) [17], [18]. If the patient was considered eligible, the same study staff asked for written informed consent and collected all required data by means of a face-to-face interview, review of clinical records or by contacting the patient's physician. Finally, the same specifically trained study staff obtained a nasopharyngeal and a pharyngeal swab from each included patient. Samples were placed in vials with viral transport medium and kept at −20°C until sent to the reference laboratory (Text S1. Laboratory procedures).

### Exclusion and inclusion criteria

Patients were excluded if they were institutionalized, non-residents in the healthcare district of enrollment, had severe egg allergy, a previous episode of laboratory-confirmed influenza in the current season or had been hospitalized in the previous 30 days.

Patients were included if they reported symptoms of influenza-like illness (ILI) [24], defined as at least one of four systemic symptoms (fever or feverishness, malaise, headache, myalgia) and one of three respiratory symptoms (cough, sore throat, shortness of breath) within 7 days of admission. Sudden onset of symptoms was not required for inclusion, and finally, patients were included if they belonged to targeted groups for influenza vaccination as defined in Valencia because of concurrent co-morbidity or age 60 and over [23].

### Comparison groups and covariates

Influenza-positive admissions were compared with influenza-negative admissions [20]–[22]. Admissions were considered as influenza-positive or influenza-negative according to rtRT-PCR results (Text S1. Laboratory procedures).

Information was obtained on age, sex, eligibility criteria, hospitalization date, time from symptoms onset to hospitalization, time to swabbing, presence of major underlying medical conditions, long-term treatments, pregnancy status, number of physician encounters in the last three months, number of hospitalizations in the last year, prescription of antivirals, smoking habits, intensive care unit admission, death in hospital, length of stay, and three first recorded diagnoses at discharge or at death in hospital. The Barthel index was obtained in study subjects aged 65 or over [25]. Social class was assigned according to occupation [26], [27].

### Vaccination status

Reports of influenza vaccination were obtained by direct questioning and independently by a researcher blinded to patient characteristics through consultation of the Valencia population-based Vaccine Information System (Vaccine Information System), from which vaccination date, vaccine type, batch number, and manufacturer were ascertained. We obtained Vaccine Information System data on influenza vaccination for the 2010–2011 and 2009–2010 influenza seasons, monovalent 2009 pandemic, and 23 polysaccharide plain pneumococcal vaccine (23PPV).

A patient was considered immunized with the 2011–2012 influenza seasonal vaccine if the vaccine was recorded in the Vaccine Information System as administered more than 14 days before the date of the ILI onset or if the patients reported vaccination more than two weeks before symptoms onset.

### Statistical analysis

We compared the differences in distribution using the Fisher exact test, Pearson chi square, Student's t-test or Kruskal-Wallis test, depending on the nature of the variable. We ascertained the contribution of covariates to the risk of laboratory confirmed influenza using the likelihood-ratio test (LRT).

We assessed confounding by considering biological and epidemiological plausibility and by analysis of the crude odds ratio of being a case and vaccinated compared to the same odds ratio when adjusted, one by one, by the collected covariates. We similarly estimated the LRT for departure from a linearity (P<0.05) in categorical ordered variables and the presence of interaction between potential confounders and current season vaccination. Evidence of clustering by enrollment site or epidemiological week was estimated by fitting random effects logistic regression models of the odds ratio of being influenza-positive and vaccinated.

### Influenza vaccine effectiveness

IVE was defined as 100 x (1 - odds ratio). The odds ratio was estimated using a test-negative approach, comparing the odds of vaccination in admissions with confirmed influenza with the odds of vaccination in influenza-negative admissions [20]–[22]. The adjusted odds ratio (OR) was obtained with adjusted multivariate multilevel random effects logistic regression models, according to evidence on confounding, linearity, interaction, or clustering.

We defined six groups for IVE estimation: a) all subjects, b) subjects aged under 65, c) subjects aged 65 or older, d) subjects aged 65 or older targeted for vaccination with the intradermal vaccine, e) subjects aged 65 or older targeted for vaccination with the virosomal vaccine, and f) subjects aged under 65 targeted for vaccination with the classical IM split influenza vaccine.

The by type of vaccine estimates were restricted to subjects for whom vaccine type was ascertained through Vaccine Information System consultation. For groups “d” (intradermal) and “e” (virosomal) the analyses were restricted to residents of the health districts where the type of vaccine was distributed. Patients registered as vaccinated with vaccines not recommended in their district of residence or vaccinated with vaccines recommend for a different age group were not included in the analysis of IVE by type of vaccine.

All IVE analyses accounted for calendar time defined by epidemiological week of admission and hospital of enrollment, and were restricted to weeks in which at least two influenza-related admissions were observed. The same criteria were applied to the subgroup analyses. Given that this was a predominant A(H3N2) season and that only 5 out of 549 samples positive for influenza were not A(H3N2) we limited our analysis to the 544 samples positive for influenza A(H3N2).

All probabilities were 2-tailed; *P*<0.05 was considered significant. Associations were considered statistically significant when the 95% confidence interval (CI) of the OR did not include the unit. Analyses were performed with Stata 12.1 (StataCorp, College Station, TX).

## Results

**Figure 1:**
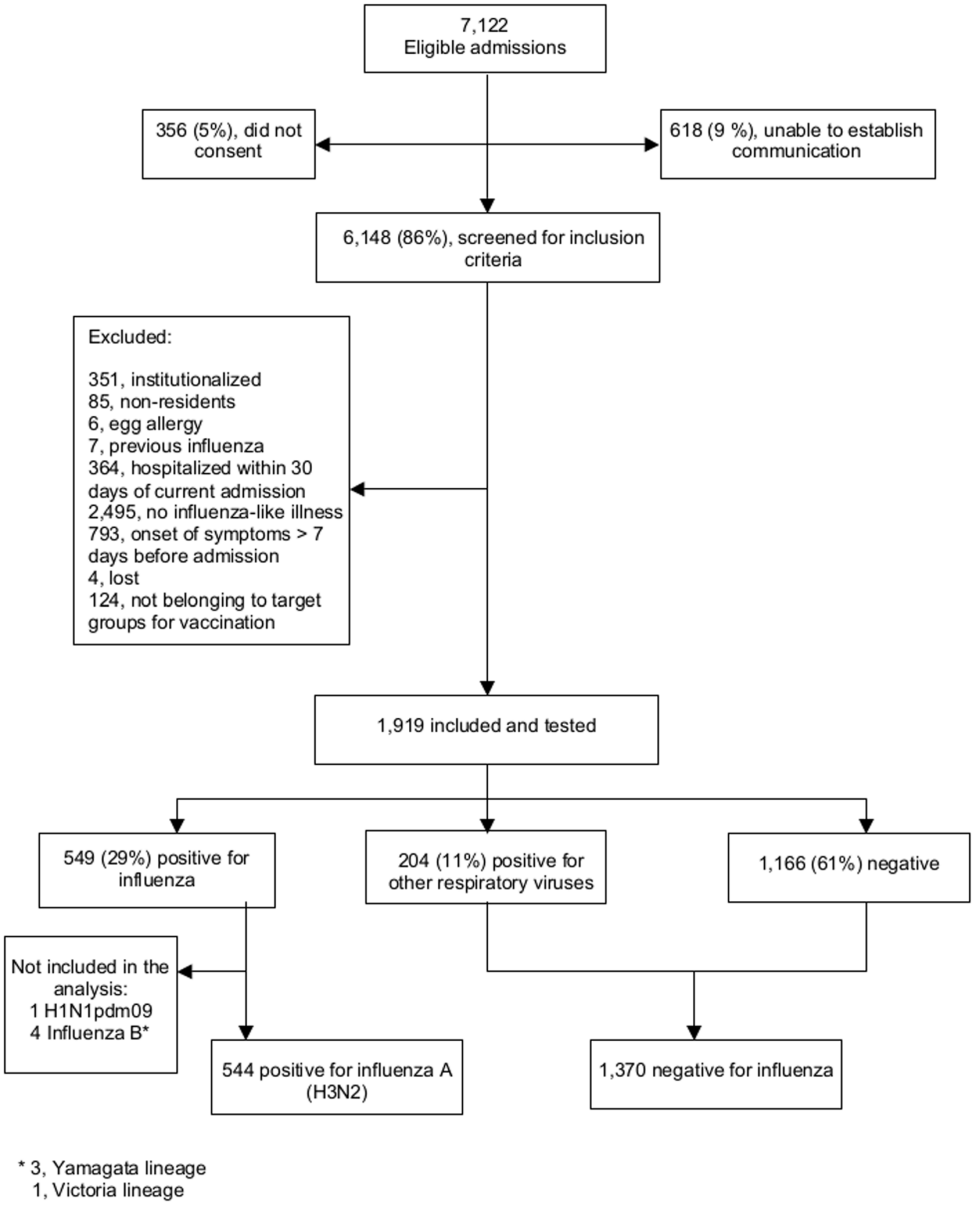
Flow diagram of study subjects.

**Figure 2:**
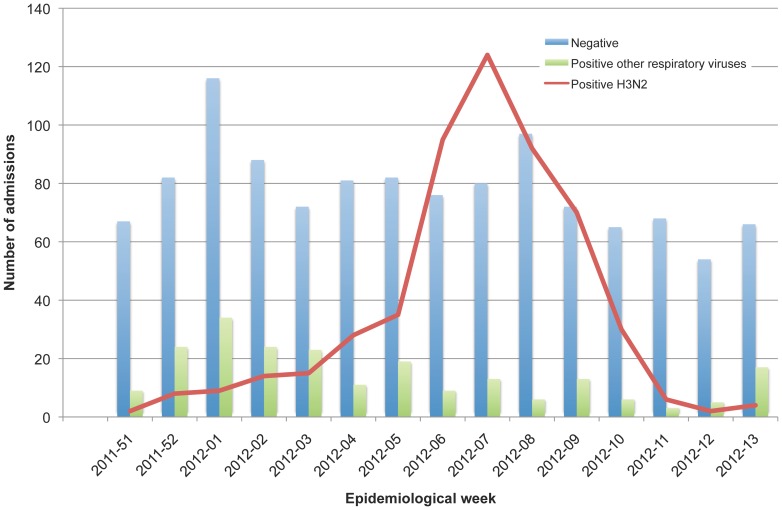
Admissions by epidemiological week and laboratory result.

We identified 7,122 eligible admissions and included after consent 1,914 subjects who complied with the criteria for inclusion. A total of 544 (29%) were positive for influenza A(H3N2) and 1,370 (71%) were negative for influenza (Figure 1). The study period began on December 18, 2011, epidemiological week 51–2011, and ended on March 31, 2012, epidemiological week 13–2012 (Figure 2).

### Subject characteristics and clinical outcomes according to PCR result for influenza

Influenza-positive subjects were admitted more often because of acute respiratory conditions (Table 1). Cough and fever were significantly more common in influenza-positive subjects than in negative subjects (Table 1). There were no differences in either length of stay or death while at hospital, and intensive care unit admission was slightly more frequent in negatives subjects (Table 1). Positive subjects did not differ from negative subjects on age, sex, body mass index or smoking habits. Partially skilled and unskilled employment types were overrepresented among positives (Table 2). The distribution of high-risk conditions was similar among positive and negative subjects (Table 2). Negative subjects consulted their general practitioner and had been hospitalized in the last 12 months more often than the positive subjects (Table 2). In subjects aged 65 or older moderate to severe functional impairment (Barthel score below 60) was more frequent in negative subjects compared with positive subjects (22% compared to 16%; *P* = 0.0133).). Finally, we obtained swabs within seven days of ILI onset in 93% influenza-positive admissions compared to with 87% of negative admissions (Table 3) without evidence of a trend in positive results according to the elapsed days (*P* = 0.1764).

**Table 1:** Main complaint at admission, influenza-like-illness symptoms within seven days to admission and clinical outcomes in influenza positive compared to influenza negative admissions

		Positive for influenza A(H3N2)	Negative for influenza	Crude odds ratio	P-value
		n = 544	n = 1370	(95% CI)	(LR test)
		Number (%)	Number (%)		
Main complaint at admission					
	Respiratory	446 (82)	1019 (74)	1.6 (1.2–2.0)	0.0003
	Cardiovascular	10 (2)	85 (6)	0.3 (0.4–0.6)	<0.0001
	Sepsis, SIRS, metabolic failure	86 (16)	238 (17)	0.9 (0.7–1.2)	0.4081
	Confusion, convulsion	1 (0.2)	13 (1)	0.2 (0.0–1.5)	0.0448
	Myalgias	1 (0.2)	15 (1)	0.2 (0.0–1.3)	0.0239
Influenza-like-illness symptoms		Number (%)	Number (%)		
	Sudden onset	328 (60)	792(58)	1.1 (0.9–1.4)	0.3192
	Fever	468 (86)	1030(75)	2.0 (1.6–2.7)	<0.0001
	Myalgia-malaise or headache	425 (78)	1064 (78)	1.0 (0.8–1.3)	0.8267
	Cough	508 (93)	1180 (86)	2.3 (1.6–3.3)	<0.0001
	Sore throat	178 (33)	416 (30)	1.1 (0.9–1.4)	0.3164
	Dyspnea	496 (91)	1270 (93)	0.8 (0.6–1.2)	0.2659
Length of stay (days), mean (SD)		7.3 (5.6)	7.3 (6.0)		0.9278
Intensive care unit admission					
	No	535 (98)	1325 (97)	1.0	0.0408
	Yes	9 (2)	45 (3)	0.5 (0.2–1.0)	
Died during hospital stay					
	No	513 (94)	1289 (94)	1.0	0.8569
	Yes	31 (6)	81 (6)	1.0 (0.6–1.5)	

CI Confidence Interval. LR Likelihood ratio. SD (standard deviation).

SIRS: Systemic inflammatory response syndrome.

Metabolic failure: hyperglycemic or hypoglycemic commas, acute renal failure, and disorders of fluid, electrolyte and acid-base balance.

**Table 2:** Characteristics of study subjects according to PCR result.

		Positive for influenza A(H3N2)	Negative for influenza	Crude odds ratio	P-value
		n = 544	n = 1370	(95% CI)	(LR test)
		Number (%)	Number (%)		
Age (in years) group					
	18–49	23 (4)	64 (5)	1.0	0.0908
	50–64	63 (12)	187 (14)	0.9 (0.5–1.6)	
	65–74	107 (20)	302 (22)	1.0 (0.6–1.7)	
	75–79	122 (22)	247 (18)	1.4 (0.8–2.3)	
	80–84	109 (20)	232 (17)	1.3(0.8–2.2)	
	> = 85	120 (22)	338 (25)	1.0 (0.6–1.7)	
Sex					
	Male	317 (58)	753 (55)	1.0	0.1880
	Female	227 (42)	617 (45)	0.9 (0.7–1.1)	
Number of high-risk conditions					
	None	76 (14)	157 (12)	1.0	0.1476
	One	199 (37)	475 (35)	0.9 (0.6–1.2)	
	Two or more	269 (50)	738 (54)	0.8 (0.6–1.0)	
Body mass index					
	<18.5	6 (1)	24 (2)	0.71 (.3–1.8)	
	18.5 to 24.9	150 (28)	427 (31)	1.0	0.1329
	25 to 29.9	234 (43)	512 (37)	1.3 (1.0–1.7)	
	30 to 39.9	139 (26)	368 (27)	1.1 (0.8–1.4)	
	> = 40	15 (3)	39 (3)	1.1 (0.0-2-0)	
Smoker					
	Never	253 (47)	620 (45)	1.0	0.1450
	Ex-smoker	211 (39)	586 (43)	0.9 (0.7–1.1)	
	Current smoker	80 (15)	164 (12)	1.2 (0.9–1.6)	
Socioeconomic class					
	Professional to skilled manual	108 (20)	339 (25)	1.0	0.0210
	Partially skilled to unskilled	436 (80)	1031 (75)	1.3 (1.0–1.7)	
Outpatient visits last three months					
	None	132 (24)	292 (21)	1.0	0.0117
	One	131 (24)	269 (20)	1.1 (0.8–1.4)	
	Two or more	281 (52)	809 (59)	0.8 (0.6–1.0)	
Number of hospitalization previous 12 months					
	None	376 (69)	870(64)	1.0	0.0008
	One	123 (23)	298 (22)	1.0 (0.7–1.2)	
	Two	26 (5)	100 (7)	0.6 (0.4–0.9)	
	Three or more	19 (3)	102 (7)	0.4 (0.3–0.7)	

PCR real-time reverse transcription-polymerase chain reaction. CI Confidence Interval. LR Likelihood ratio.

**Table 3:** Time since symptom onset to swabbing according to PCR result.

		Positive for influenza A(H3N2)	Negative for influenza	Crude odds ratio	P-value ^a^
		n = 544	n = 1370	(95% CI)	
		Number (%)	Number (%)		
Onset to swab (days)					
	1 to 2	77 (14)	271 (20)	1.0	<0.1764
	3 to 4	237 (44)	440 (32)	1.9 (1.4–2.6)	
	5 to 7	194 (36)	486 (35)	1.4 (1.0–1.9)	
	>7	36 (7)	173 (13)	0.7 (0.5–1.1)	

PCR real-time reverse transcription-polymerase chain reaction. CI Confidence Interval.

a: Score test for trend of odds.

### Vaccination

The record of previous vaccination (influenza or other) in the Vaccine Information System was similar in both groups (Table 4). Overall, 1,086 (57%) subjects had a record of vaccination with the 2011–2012 seasonal influenza vaccine 15 or more days before symptoms onset, whereas 1,134 (59%) subjects reported being vaccinated for influenza two weeks or more before ILI symptoms onset. We considered as immunized 58% of influenza-positive and 62% of influenza-negative (*P* = 0.0585) admissions (Table 4).

**Table 4:** Vaccination according to PCR result.

		Positive for influenza A(H3N2)	Negative for influenza	Crude odds ratio	P-value
		n = 544	n = 1370	(95% CI)	(LR test)
		Number (%)	Number (%)		
Included in Vaccine Information System a					
	No	73 (13)	218 (16)	1.0	0.1662
	Yes	471 (87)	1,152 (84)	1.2 (0.9–1.6)	
Immunized with current 2011–2012 season influenza vaccine ^b^					
	No	230 (42)	515 (38)	1.00	0.0585
	Yes	314 (58)	855 (62)	0.82 (0.67–1.01)	
Days since vaccination to onset of symptoms c					
	Mean (s.d.)	119 (21)	109 (32)		<0.0001
	14 to 89	25 (9)	243 (31)	1.0	<0.0001
	90 to 119	105 (37)	358 (32)	4.0 (2.5–6.3)	
	120–184	158 (55)	297 (37)	5.2 (3.3–8.2)	
Vaccinated on previous seasons					
Seasonal, 2010–2011					
	No	239 (44)	558 (41)	1.0	0.2003
	Yes	305 (56)	812 (59)	0.9 (0.7–1.1)	
Seasonal, 2009–2010					
	No	223 (41)	547 (40)	1.0	0.6682
	Yes	321 (59)	823 (60)	1.0 (0.8–1.2)	
Pandemic, 2009–2010					
	No	377 (69)	964 (70)	1.0	0.6473
	Yes	167 (31)	406 (30)	1.1 (0.9–1.3)	
23 polysaccharide pneumococcal vaccine					
	No	392 (72)	1,067 (78)	1.0	0.0076
	Yes	152 (28)	303 (22)	1.4 (1.1–1.7)	

PCR real-time reverse transcription-polymerase chain reaction. CI Confidence Interval. LR Likelihood ratio.

a Any vaccination ever recorded in the Vaccine Information System.

b. A patient was considered immunized with the 2011–2012 influenza seasonal vaccine if the vaccine was recorded in the Vaccine Information System as administered more than 14 days before the date of the ILI onset or if the patients reported vaccination more than two weeks before symptoms onset.

c Data from 1,086 (288 positive and 798 negative) patients vaccinated 15 or more days before onset of symptoms according to the information in the Vaccine Information System out of 1,169 classified as immunized because of information retrieved from the Vaccine Information System or by patient's recall of vaccination two weeks before symptoms onset.

The mean (standard deviation) number of days from vaccination to onset of symptoms for influenza-positive subjects was 119 (21) compared with 109 (32) for influenza-negative subjects, with a significant trend for increasing risk (*P*<0.0001) of a positive influenza result in the unadjusted analysis considering days elapsed since vaccination to onset of symptoms (Table 4). This effect was no longer observed after adjusting by epidemiological week (Figure S1).

According to the Vaccine Information System similar proportions of positive and negative subjects were vaccinated with the influenza vaccine in the previous two seasons (2010–2011, 2009–2010) or with the monovalent pandemic 2009 H1N1vaccine (Table 4). The positive subjects were vaccinated with the 23PPV more often than negative subjects.

### Type of vaccine

The intradermal vaccine was administered to 60% of influenza-positive and 70% of influenza-negative admissions aged 65 or over (*P* = 0.0080). The virosomal vaccine was administered to 68% of influenza-positive and 68% of influenza-negative admissions aged 65 or over (*P* = 0.8970). The split classical IM influenza vaccine was administered to 20% of the positive and 46% of the negative (*P* = 0.0050) subjects aged 18 to 64 (Table 5 and Table S3).

**Table 5:** Influenza vaccines recorded as administered by type of vaccine, age group, and PCR result.

	PCR result for influenza						
Type of vaccine ^a^	Positive		Negative		Total		P value
	n	%	n	%	n	%	
Intradermal (subjects> = 65 years of age) ^b^							
No	82	40.0	136	29.6	218	32.8	
Yes	123	60.0	324	70.4	447	67.2	*P = 0.0080*
Total	205	100.0	460	100.0	665	100.0	
Virosomal (subjects> = 65 years of age) ^b^							
No	60	32.4	102	31.9	162	32.1	
Yes	125	67.6	218	68.1	343	67.9	*P = 0.8970*
Total	185	100.0	320	100.0	505	100.0	
Split conventional (subjects 18 to <65 years of age) ^c^							
No	39	79.6	30	53.6	69	65.7	
Yes	10	20.4	26	46.4	36	34.3	*P = 0.0050*
Total	49	100.0	56	100.0	105	100.0	

PCR real-time reverse transcription-polymerase chain reaction.

a. Recorded vaccinations in the Vaccine Information System only (see Table 4 for the percentage of patients included in the Vaccine Information System). The percentages reported over all participants included in the Vaccine Information System and with any vaccination ever recorded in the Vaccine Information System.

b. Encompassing epidemiological weeks 52 to 12, with influenza related admissions identified in> = 65 years subjects. This was the age group targeted to receive this type of vaccine.

c. Encompassing epidemiological weeks 4 to 10, with influenza related admissions identified in <65 years old subjects. This was the age group targeted to receive this type of vaccine.

### Effect modification, confounding and clustering

There was moderate evidence of age in deciles acting as an effect modifier, *P* = 0.0540 (Figure S2). Outpatient consultations in the preceding three months and previous vaccination with the 23PPV were associated with both being positive for influenza and current season influenza vaccination (Table 1, Table S4a and Table S4b). Overall, there was a strong (*P*<0.0001) clustering effect due to epidemiological week and to hospital.

### Influenza vaccine effectiveness

**Figure 3:**
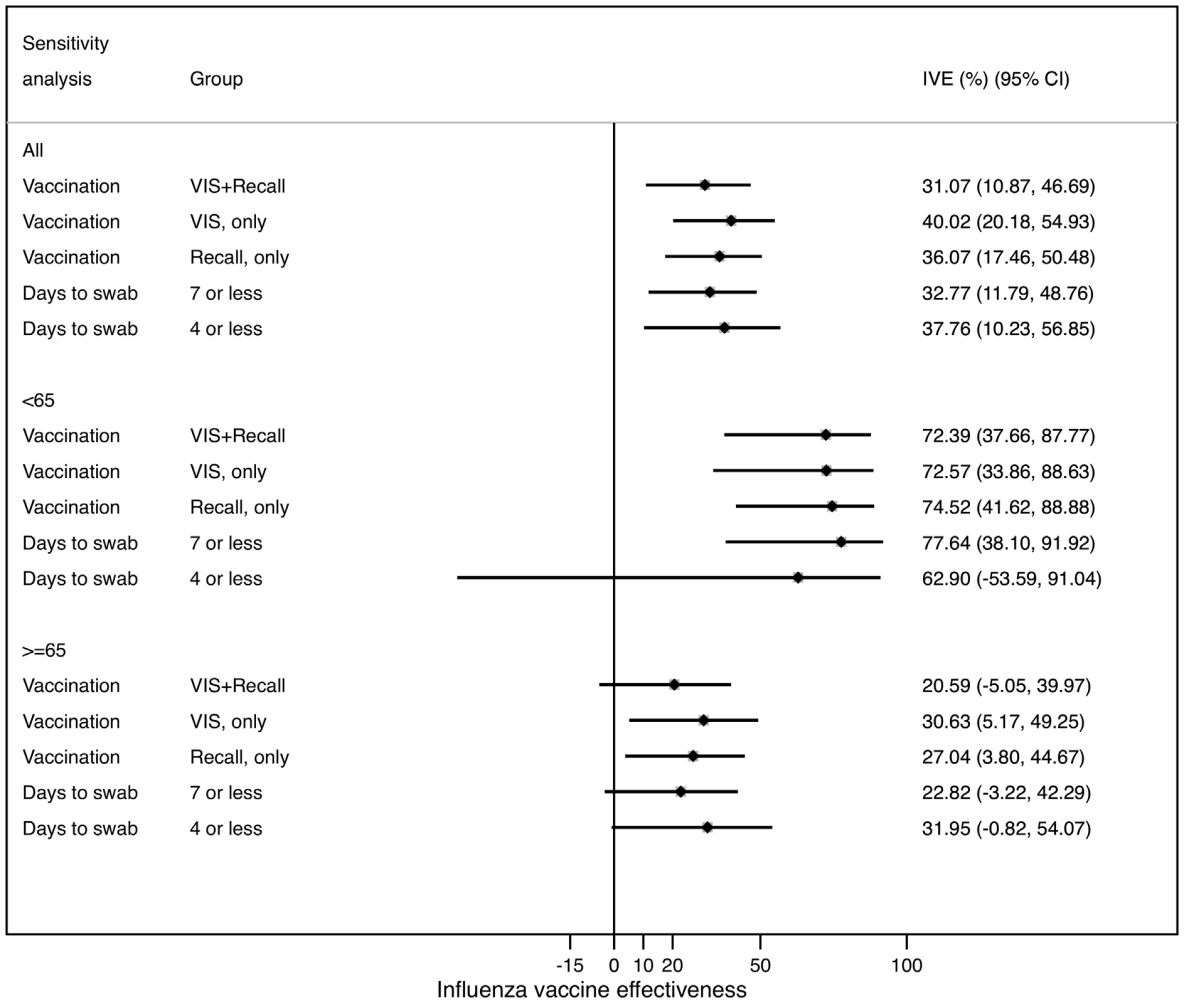
Sensitivity analysis of adjusted influenza vaccine effectiveness. Adjusted influenza vaccine effectiveness (IVE) assessed for all ages, <65 and> = 65, and by: a) vaccination ascertainment method: Vaccine Information System (VIS), as reported by the patient (recall) and both combined; b) days to swab: seven or less, four or less. Reference category for all analyses includes all patients irrespective of time elapsed to swab and vaccination according to VIS or recall. OR: adjusted odds ratio. OR adjusted as reported in footnotes in Table 6.

The overall adjusted IVE against hospitalization with PCR-confirmed influenza was 31% (95%CI, 11% to 47%) (Table 6). The IVE estimate in subjects aged under 65 was 72% (38% to 88%) and 21% (−5% to 40%) for those aged 65 or older. Those estimates did not vary when we conducted sensitivity analyses taking into account how vaccination was defined (registry and recall, registry only, or recall only) or elapsed time from symptoms onset to swabbing (irrespective of elapsed time, seven days or less, or four days or less) (Figure 3). Overall the results were homogenous irrespective of how vaccination exposure was defined or the cut-off for days elapsed since symptoms onset to swabbing. There was strong evidence of heterogeneity (I-squared test, 74%) by age. The heterogeneity inside the groups shown in Figure 3 was 0%.

**Table 6:** Influenza vaccine effectiveness in preventing admissions related to H3N2 influenza.

Considered groups	Vaccinated positives (n)/all positives (N)		Vaccinated negatives (n)/all negatives (N)		IVE (IC95%)		IVE (95% CI)	
	n/N (%)		n/N (%)		Non-adjusted		Adjusted	
Overall	314/544	(58)	855/1370	(62)	17.8	(−0.7 to 32.8)	31.1	(10.9 to 46.7) ^a^
By age group, cut-point at 65 years of age								
<65	20/82	(24)	50/102	(49)	66.5	(35.1 to 82.6)	72.4	(37.7 to 87.8) ^b^
> = 65	294/457	(64)	705/1054	(67)	10.7	(−12.4 to 29.1)	20.6	(−5.1 to 40.0) ^a^
By vaccine type, recommended age group								
Split classical IM, <65	10/49	(20)	26/56	(46)	70.4	(26.5 to 88.1)	67.5	(11.8 to 88.1)^ c^
Split intradermal, ≥ 65	123/205	(60)	324/460	(70)	37.0	(11.0 to 55.5)	38.9	(10.8 to 58.1) ^d^
Subunit virosomal, ≥65	125/185	(68)	218/320	(68)	2.5	(−43.6 to 33.9)	15.7	(−39.0 to 48.8)^e^

Positives admissions with influenza confirmed influenza by real time reverse transcription polymerase chain reaction (rtRT-PCR). Negatives admissions with a negative rtRT-PCR result for influenza. IVE influenza vaccine effectiveness. CI Confidence Interval.

a Strong evidence (*P*<0.002) of between hospital and between epidemiological week variability. Hospital and epidemiological week included as random effect parameters in the multilevel analysis to estimate influenza vaccine effectiveness adjusted by age (deciles), sex, number of outpatient consultations in the last three months, and previous administration of plain 23 polysaccharide pneumococcal vaccine.

b Moderate evidence of between hospital variability (*P* = 0.045) and of between epidemiological week variability (*P* = 0.015). Hospital and epidemiological week included as a random effect parameter in the multilevel analysis to estimate influenza vaccine effectiveness in this group, adjusted as in (a).

c Weak evidence of between hospital variability (*P*>0.05). Strong evidence (*P* = <0.001) of between epidemiological week variability. Epidemiological week included as a random effect parameter in the multilevel analysis to estimate split conventional influenza vaccine effectiveness in this age group, adjusted as in (a) with hospital included in the model as an indicator variable.

d Weak evidence of between hospital variability (*P* = 0.497). Strong evidence (*P* = <0.001) of between epidemiological week variability. Epidemiological week included as a random effect parameter in the multilevel analysis to estimate influenza vaccine effectiveness in this group, adjusted as in (a) with hospital included in the model as an indicator variable.

e Moderate evidence (*P* = 0.042) of between hospital variability and strong evidence (*P* = <0.001) of between epidemiological week variability. Hospital and epidemiological included as random effect parameters in a multilevel analysis to estimate influenza vaccine effectiveness adjusted as in (a).

By type of vaccine, the split classical IM influenza vaccine used in those aged 18 to under 65 targeted for influenza had an adjusted IVE against hospitalization with PCR-confirmed influenza of 68% (12% to 88%). The intradermal vaccine, used in subjects aged 65 or older in five of the healthcare districts included in the study, had an adjusted IVE against hospitalization with PCR-confirmed influenza of 39% (11% to 58%); finally, for the virosomal vaccine, used in the remaining four healthcare districts studied, the adjusted IVE against hospitalization with PCR-confirmed influenza was 16% (−39% to 49%) effectiveness (Table 6).

## Discussion

The overall adjusted estimate of IVE against laboratory-confirmed influenza A(H3N2) hospitalizations in adults targeted for vaccination in Valencia (Spain) of 31.1% (95% CI, 10.9% to 46.7%) suggested that vaccination may have prevented over 30% of the severe cases of influenza requiring hospitalization in vaccinated patients. These estimates of protection were larger in patients 18 to 64 years of age (72.4%, 95% CI, 37.7% to 87.8%) compared with patients of 65 years and older (20.6%, −5.1% to 40.0%). A classical IM vaccine used to vaccinate subjects aged under 65 displayed high IVE in preventing admissions related to influenza. In subjects aged 65 or older, the intradermal vaccine had a moderate effect in preventing admissions related to influenza. No effect was observed for the virosomal vaccine. To our knowledge, estimates on virosomal or intradermal vaccine in preventing confirmed influenza-related admissions in subjects aged 65 or older have not been published previously.

The results of observational studies are to be interpreted with caution because of the limitations related to unmeasured confounding, comparability of subjects, selection and classification bias or study design.

To take into consideration these limitations, we collected information on a comprehensive group of potential confounders that allowed us to account for frailty bias and confounding by indication [28]–[30]. Further, to improve comparability of vaccinated with unvaccinated subjects we constrained our analysis to subjects targeted for influenza vaccination.

### Selection bias

Selection bias was minimized because we enrolled most eligible patients (Figure 1), and eligibility criteria were applied with no knowledge of vaccination or influenza infection. The test-negative study design minimized selection bias, as positive and negative subjects were similar in their healthcare-seeking behavior and chances of being identified and included in the study (Text S2. Bias due to design).

### Misclassification

#### Vaccination status

Vaccination status established by interviews and by consulting vaccination records can be improved and misclassification reduced if both sources of information are combined (unpublished data). In the sensitivity analysis comparing IVE with vaccination ascertained by Vaccine Information System, by recall or with both methods combined, we obtained overlapping estimates. In the analysis by vaccine type we only included subjects with records in the Vaccine Information System information system.

#### Influenza status

We have used an endpoint of laboratory-confirmed influenza. Although it can be argued that we may have missed those influenza cases that were PCR negative at admission [15]. PCR-confirmed influenza is likely to be more specific than hospitalization with clinically diagnosed ILI, as the majority of these cases are likely to be due to other co-circulating pathogens. Mathematical modeling has suggested that false positives may lower the IVE estimates, and therefore, the use of less-specific outcomes, such as clinical ILI, may generate even less accurate IVE estimates than PCR-confirmed influenza outcome [19].

We choose not to exclude subjects according to time to swab to preserve the information provided by true influenza-positive admissions and to avoid unknown misclassification bias [31]. Supporting our decision was the absence of a trend in the percentage of positives according to the days elapsed since symptoms onset and the overlapping IVE estimates obtained in the sensitivity analysis.

### Vaccine effectiveness

Comparisons on the consistency of IVE estimates obtained in different influenza seasons or even for the same season but in different geographical locations are to be made cautiously, as IVE estimates are affected by season-to-season differences in virulence of circulating strains, levels of population immunity, healthcare-seeking behavior, matching of the vaccine recommended antigenic composition with the circulating strains, and different types of vaccines used in different settings.

Several additional reasons may explain the differences observed in point estimates of IVE (and the variability in confidence intervals) against medically attended outcomes with laboratory-confirmed H3N2 in our study compared with other test-negative studies performed during the same season [32]–[37].

First, the baseline characteristics of the patients included in the mentioned studies are different from ours in terms of risk factors that consistently affect IVE in regression analyses, as age distribution, presence of comorbidities, hospitalizations within the previous 12 months and percentage vaccinated.

In general, patients from primary-care surveillance systems, included in all of the above-mentioned studies, are younger, more likely to be free of comorbidities, and less likely to have been vaccinated or hospitalized within the previous 12 months compared with inpatients from hospital-based surveillance systems. In one study [32], patients were ascertained both at GP consultation and in hospital wards, but less than 10% of the study patients were inpatients.

Although the studies considered were performed in the northern hemisphere, differences in past exposure to H3N2 infection, infectious challenge, and vaccination policies, which are likely to vary geographically, may all underlie differences in vaccine effects across regions [38], [39].

Finally, limited sample size or sub-group analyses lead to low precision around point estimates and random highs and lows.

Despite all of these limitations, for patients aged under 65 years (Figure S3a), in terms of the confidence intervals of our results, 72% (38% to 88%) overlap with those reported in Navarre [32], 44% (−11% to 72%). For I-MOVE [33], the number is 63% (26%–82%); and for Canada [36], the number is 44%(−41% to 78%). Those results are statistically homogenous, with a pooled IVE estimate of 56%(42% to 71%). A similar result is obtained when heterogeneity among IVE estimates against laboratory-confirmed influenza is explored in IVE estimates for subjects over 50 to over 65 (Figure S3b), depending on the study [32]–[36], with a pooled IVE estimate of 30% (14 to 46%); or among studies reporting overall IVE estimates (Figure S3c) against laboratory confirmed H3N2 for the 2011–2012 season [32]–[37] with a pooled IVE estimate of 35% (26% to 44%). In summary, we report similar overall moderate to low IVE results as those reported by others for the same 2011–2012 season, with an overall better IVE in young adults belonging to target groups for vaccination and an overall lower response in the elderly.

Our estimates add to the evidence indicating a significant IVE against hospitalization with laboratory-confirmed influenza during the 2011–2012 season [33], [40]–[44]. However, these estimates vary widely across studies, which used different criteria to select the group of patients to be included in the IVE sub-group analyses. Furthermore, caution is needed because of the limited sample size usually associated with sub-group analyses, which may have led to low precision around point estimates and random highs and lows, making comparisons across studies difficult. Our moderate-low overall estimate of IVE against hospitalization for 2011–2012 is very similar to that reported by others. It has been suggested that antigenically different circulating A(H3N2) from that of the vaccine strain in Europe may explain the low effectiveness reported in Europe [45].

Influenza A (H3N2) viruses circulating in Spain during the 2011–2012 season were distributed in three genetic groups that displayed evidence of antigenic diversity regarding the A/Perth/16/2009 vaccine strain [46], [47]. The results we report for those aged 65 and over are consistent with those reported previously for the elderly in seasons with poor matching between vaccine and circulating strains [8], [48], [49].

Although we do not have information to support that mutations in the egg-passaged A/Perth/16/2009 (H3N2) NYMC X-187 derived from the A/Victoria/210/2009 strains used in the 2011–2012 vaccines could explain the moderate influenza vaccine effectiveness we have found, we cannot disregard that egg passage could have a similar effect in the immunogenic capability of the 2011–2012 vaccine as the one described for the H3N2 egg-passaged strain in the 2012–2013 seasonal vaccine [12], [45], [50].

## Conclusions

In this season we observed relevant influenza vaccine effectiveness in adults aged 18 to 64 years old in contrast to a low and non-significant vaccine effectiveness in subjects aged 65 and over.

Most of the variability in IVE estimates observed by us in the studied season could be explained by age.

The significant effect of the intradermal vaccine, recommended for subjects aged 65 and over, in a season with a questionable match between the circulating and vaccine strain, could be interpreted as a proof of concept of the opportunities of the intradermal approach to protect those more at risk because of advancing age.

The year-by-year drift of the influenza virus and the variable protection conferred by different influenza vaccine formulations, combined with the interaction of those factors with age and other risk conditions, justify the year-by-year active surveillance of severe cases of influenza and of IVE in preventing severe influenza-related outcomes as the one performed in the Valencia Hospital Network for the Study of Influenza and Respiratory Virus Disease.

## Supporting Information

Figure S1 **Risk of being influenza-positive according to time elapsed since vaccination to onset of symptoms (vaccination waning immunity effect) when not adjusting for calendar time and after adjusting for calendar time.**
(PDF)

Figure S2 **Odds ratio of being positive for influenza if vaccinated by age in deciles.**
(PDF)

Figure S3 **a**. Comparative analysis of influenza vaccine effectiveness estimates published by various groups in preventing medically attended influenza-like illness or admissions with H3N2-confirmed influenza for the 2011–2012 season in subjects aged less than 65 years of age. **b**. Comparative analysis of influenza vaccine effectiveness estimates published by various groups in preventing medically attended influenza-like illness or admissions with H3N2-confirmed influenza for the 2011–2012 season in subjects aged at least over 50 years of age. **c**. Comparative analysis of influenza vaccine effectiveness estimates published by various groups in preventing medically attended influenza-like illness or admissions with H3N2-confirmed influenza for the 2011–2012 season, all age groups.
(PDF)

Table S1 **Eligibility criteria: list of admission diagnoses possibly associated with an influenza infection.**
(DOC)

Table S2 **Influenza vaccination as recorded in the Vaccine Information System for the 2011–2012 influenza season.** Vaccine type and healthcare district/hospital.
(DOC)

Table S3 **a**. Characteristics of unvaccinated and vaccinated subjects (i); demographic, socioeconomic, smoking habits, body mass index, and number of risk factors. **b**. Characteristics of unvaccinated and vaccinated subjects (ii): use of healthcare services, previous vaccination, length of stay, intensive care unit admission and in-hospital death.
(PDF)

Text S1 **Laboratory procedures.**
(DOC)

Text S2 **Bias due to design.**
(DOC)
