# Supplementary material for: Influenza Vaccine Effectiveness in Preventing Influenza A(H3N2)-Related Hospitalizations in Adults Targeted for Vaccination by Type of Vaccine: A Hospital-Based Test-Negative Study, 2011–2012 A(H3N2) Predominant Influenza Season, Valencia, Spain
Source: PLoS One. 2014 Nov 13;9(11):e112294. doi: 10.1371/journal.pone.0112294 (PMC4230985; doi:10.1371/journal.pone.0112294)
Supplement: Figure S1 — Risk of being influenza-positive according to time elapsed since vaccination to onset of symptoms (vaccination waning immunity effect) when not adjusting for calendar time and after adjusting for calendar time. (PDF) [file pone.0112294.s001.pdf]

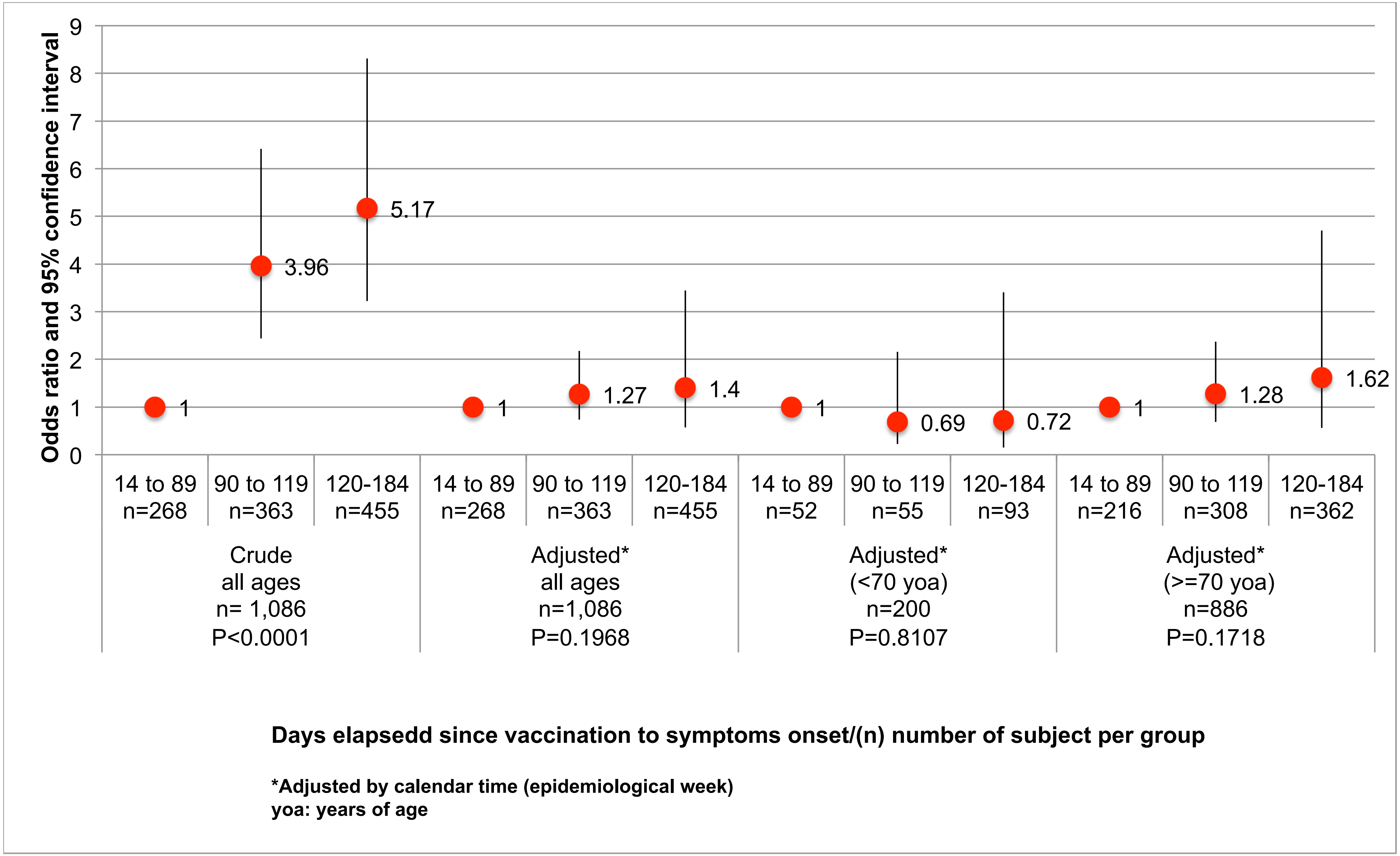
Odds ratio and 95% confidence interval
9
8
7
6
5
4
3
2
1
0
1
3.96
5.17
1
1.27
1.4
1
0.69
0.72
1
1.28
1.62
14 to 89 n=268
90 to 119 n=363
120-184 n=455
14 to 89 n=268
90 to 119 n=363
120-184 n=455
14 to 89 n=52
90 to 119 n=55
120-184 n=93
14 to 89 n=216
90 to 119 n=308
120-184 n=362
Crude all ages n= 1,086 P<0.0001
Adjusted* all ages n=1,086 P=0.1968
Adjusted* (<70 yoa) n=200 P=0.8107
Adjusted* (>=70 yoa) n=886 P=0.1718
Days elapsedd since vaccination to symptoms onset/(n) number of subject per group
*Adjusted by calendar time (epidemiological week)
yoa: years of age
