# Supplementary figures and images for: Influenza Vaccine Effectiveness in Preventing Influenza A(H3N2)-Related Hospitalizations in Adults Targeted for Vaccination by Type of Vaccine: A Hospital-Based Test-Negative Study, 2011–2012 A(H3N2) Predominant Influenza Season, Valencia, Spain
Source: PLoS One. 2014 Nov 13;9(11):e112294. doi: 10.1371/journal.pone.0112294 (PMC4230985; doi:10.1371/journal.pone.0112294)

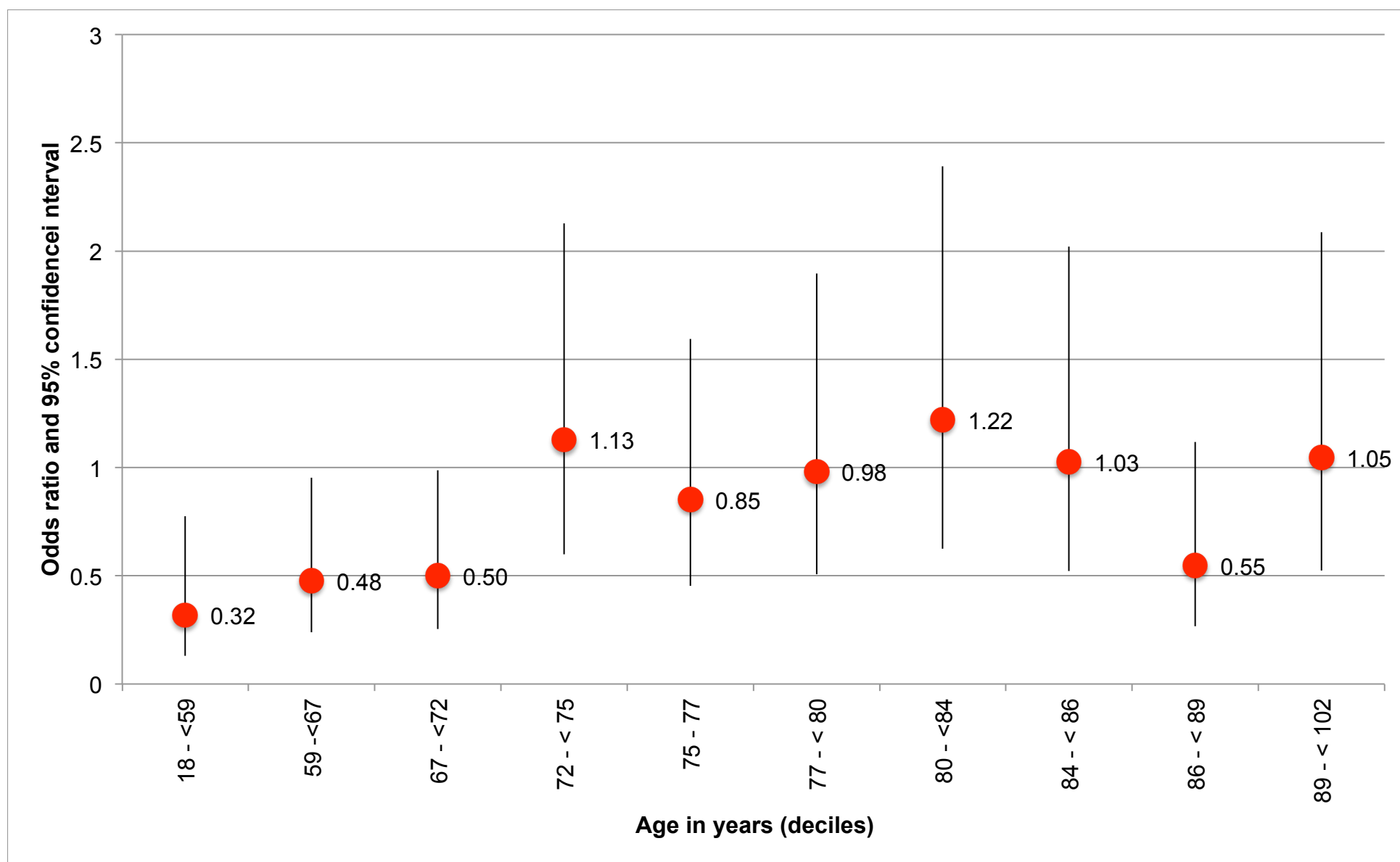

Supplement: Figure S2 — Odds ratio of being positive for influenza if vaccinated by age in deciles. (PDF) [file pone.0112294.s002.pdf]
