# Supplementary material for: Influenza Vaccine Effectiveness in Preventing Influenza A(H3N2)-Related Hospitalizations in Adults Targeted for Vaccination by Type of Vaccine: A Hospital-Based Test-Negative Study, 2011–2012 A(H3N2) Predominant Influenza Season, Valencia, Spain
Source: PLoS One. 2014 Nov 13;9(11):e112294. doi: 10.1371/journal.pone.0112294 (PMC4230985; doi:10.1371/journal.pone.0112294)
Supplement: Figure S3 — a. Comparative analysis of influenza vaccine effectiveness estimates published by various groups in preventing medically attended influenza-like illness or admissions with H3N2-confirmed influenza for the 2011–2012 season in subjects aged less than 65 years of age. b. Comparative analysis of influenza vaccine effectiveness estimates published by various groups in preventing medically attended influenza-like illness or admissions with H3N2-confirmed influenza for the 2011–2012 season in subjects aged at least over 50 years of age. c. Comparative analysis of influenza vaccine effectiveness estimates published by various groups in preventing medically attended influenza-like illness or admissions with H3N2-confirmed influenza for the 2011–2012 season, all age groups. (PDF) [file pone.0112294.s003.pdf]

Supporting Figure S3

Puig-Barberà et al., Influenza vaccine effectiveness in preventing influenza A(H3N2)-related hospitalizations in adults targeted for vaccination by type of vaccine: a hospital-based test-negative study, 2011-2012 A(H3N2) predominant influenza season, Valencia, Spain, *PLoS One, 2014*

Correspondence:

Dr. Joan Puig-Barberà,
Vaccines Research Area,
Fundación para el Fomento de la Investigación Sanitaria y Biomédica de la Comunitat Valenciana (FISABIO),
Avenida Cataluña, 21 46020 Valencia, Spain.
Phone: +34 961 925 948 Fax: +34 961 925 938
E-mail: puig_joa@gva.es

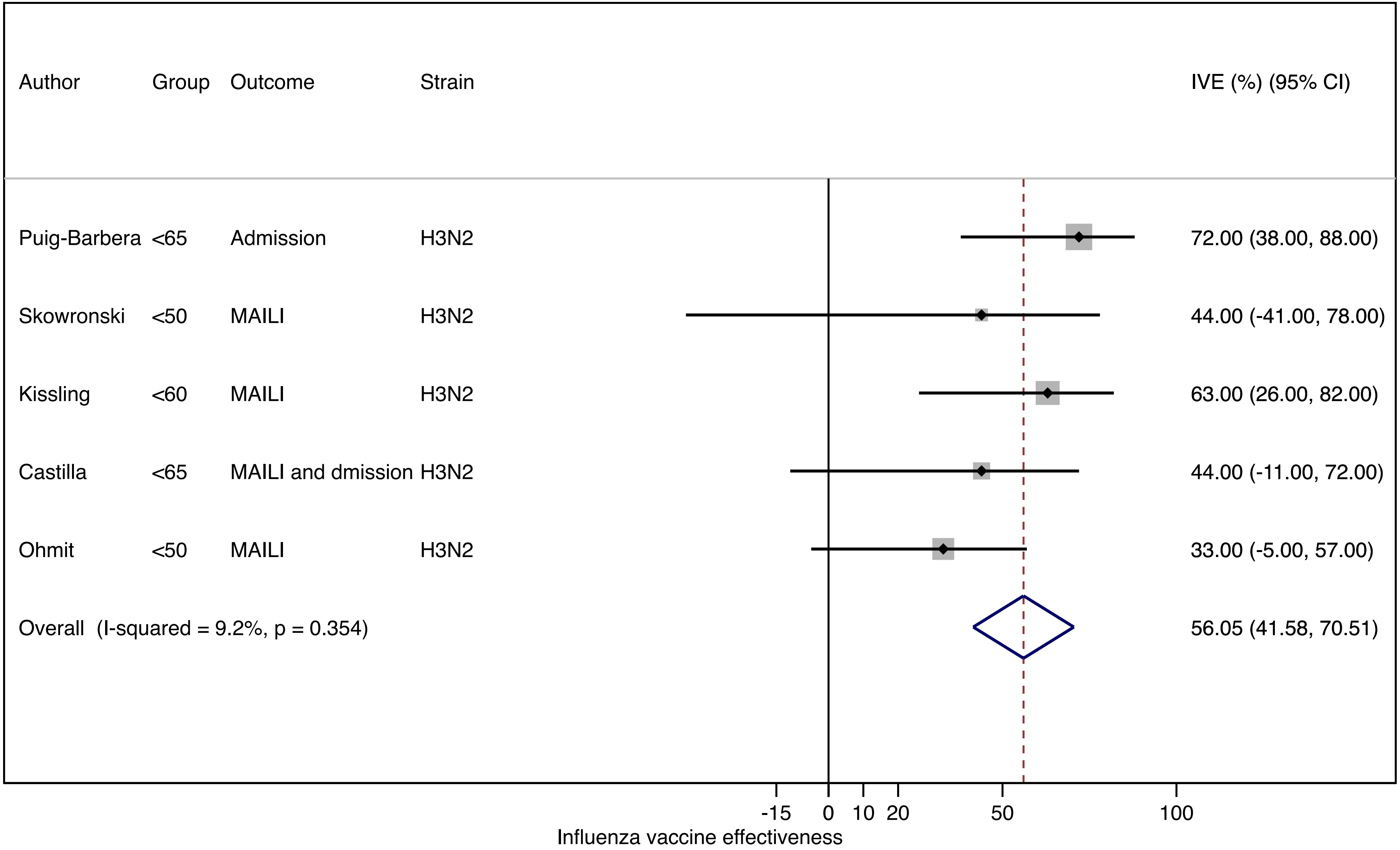

Figure S3a

MAILI: Medically attended influenza like illness or acute respiratory infection

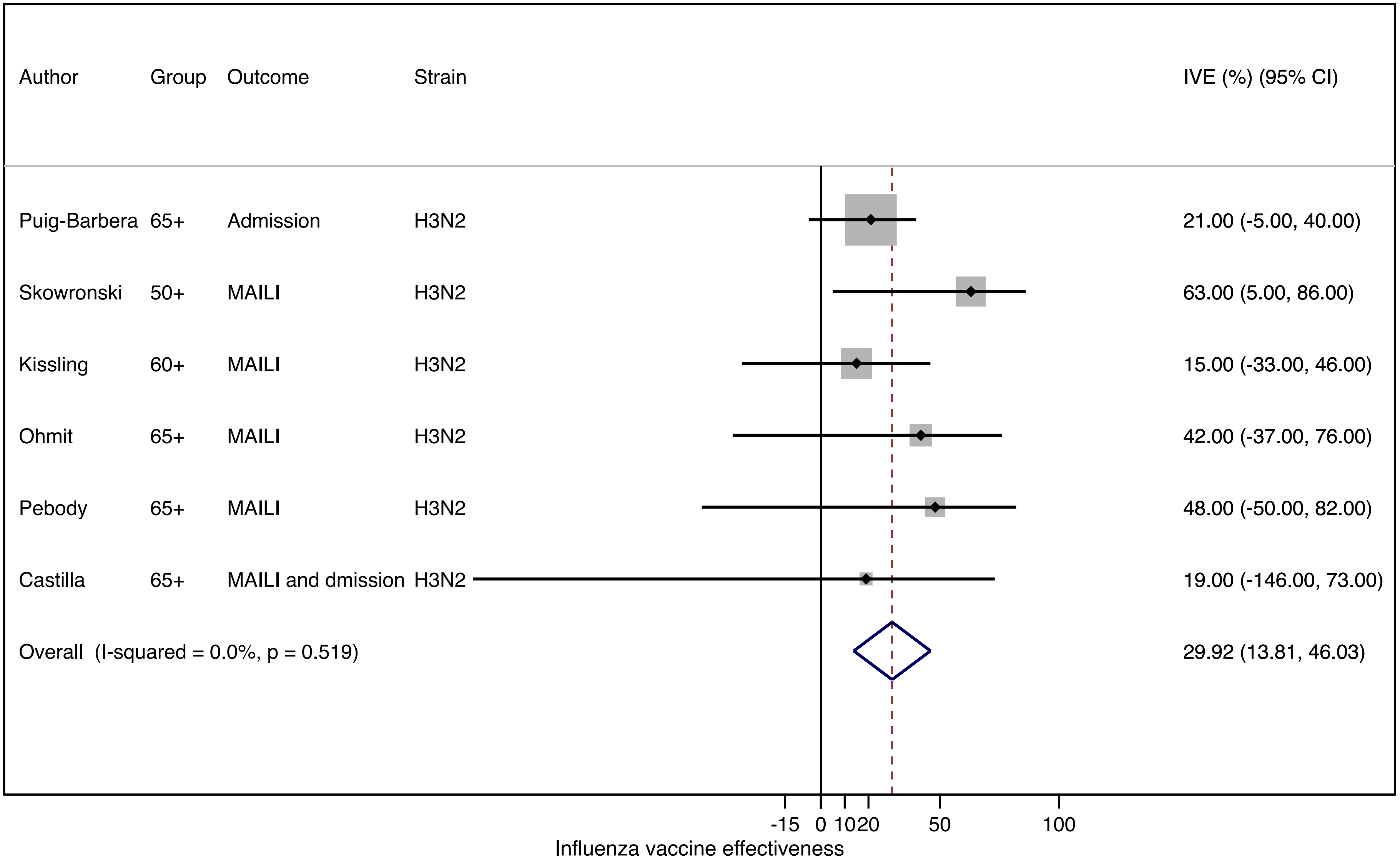

Figure S3b

MAILI: Medically attended influenza like illness or acute respiratory infection

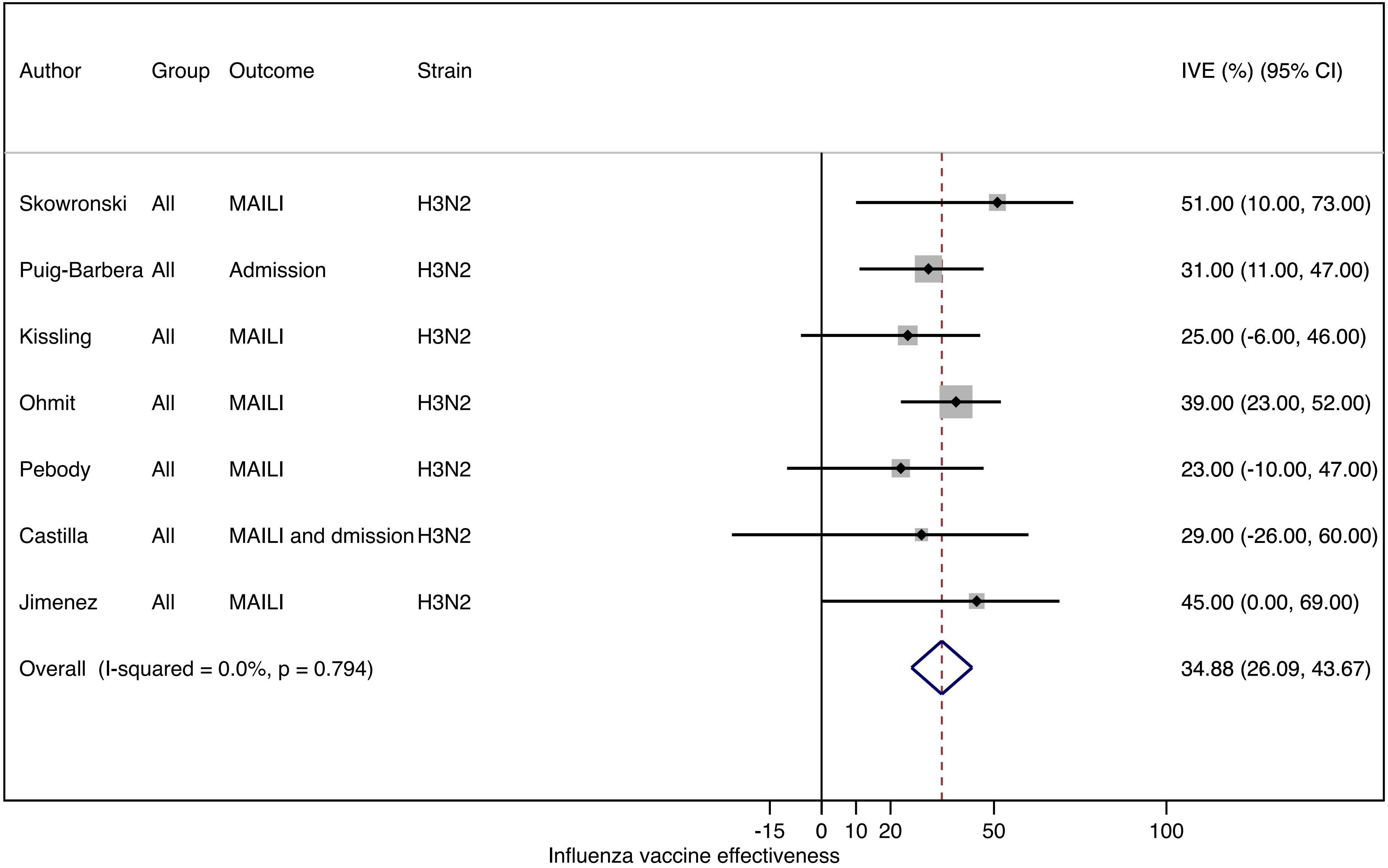

Figure S3c

MAILI: Medically attended influenza like illness or acute respiratory infection
