# Supplementary material for: Influenza Vaccine Effectiveness in Preventing Influenza A(H3N2)-Related Hospitalizations in Adults Targeted for Vaccination by Type of Vaccine: A Hospital-Based Test-Negative Study, 2011–2012 A(H3N2) Predominant Influenza Season, Valencia, Spain
Source: PLoS One. 2014 Nov 13;9(11):e112294. doi: 10.1371/journal.pone.0112294 (PMC4230985; doi:10.1371/journal.pone.0112294)
Supplement: Table S1 — Eligibility criteria: list of admission diagnoses possibly associated with an influenza infection. (DOC) [file pone.0112294.s004.doc]

Table S1. Eligibility criteria: list of admission diagnoses that were used to indentify emergency admissions as possibly associated with an influenza infection and considered for inclusion.

| Criteria | ICD 9 Codes | ICD 10 Codes |
| --- | --- | --- |
| Acute respiratory infection | 382.9; 460-466 | J00-J06, J20-J22, H66.90 |
| Acute myocardial infarction or acute coronary syndrome | 410-411 and 413-414 | I20-I25.9 |
| Asthma | 493-493.92 | J45.2-J45.22, J45.9-J45.998, J44-J44.9 |
| Heart failure | 428-429.0 | I50-I50.9; I51.4 |
| Pneumonia and influenza | 480-488 | J09-J18 |
| Chronic Pulmonary Obstructive disease | 490, 491, 492, 496 | J40-J44.9 |
| Myalgia | 729.1 | M79.1 |
| Metabolic failure (diabetic coma, renal dysfunction, acid-base disturbances, alterations to the water balance) | 250.1- 250.3; 584-586; 276-277 | E11.9, E10.9, E11.65, E10.65, E10.11, E11.01, E10.641, E11.641, E10.69, E11.00, E10.10, E11.69, N17.0, N17.1, N17.2, N17.8, N17.9, N18.1, N18.2, N18.3, N18.4, N18.5, N18.6M N18.9, N19, E87.0, E87.1, E87.2, E87.3, E87.4, E87.5, E87.6, E87.70, E87.71, E87.79, E86.0, E86.1 |
| Altered consciousness, convulsions, febrile-convulsions | 780.01-780.02; 780.09; 780.31-780.32 | R40.20, R40.4, R40.0, R40.1, R56.00, R56.01 |
| Dyspnea/respiratory abnormality | 786.0 | R06.0, R06-R06.9 |
| Respiratory abnormality | 786.00 | R06.9 |
| Shortness of breath | 786.05 | R06.02 |
| Respiratory abnormality nec | 786.09 | R06.3, R06.00, R06.09, R06.83 |
| Respiratory symptoms/chest symptoms | 786.9 | R06.89 |
| Fever or fever unknown origin or non specified | 780.6-780.60 | R50, R50.9 |
| Cough | 786.2 | R05 |
| Sepsis, Systemic inflammatory response syndrome | 995.90-995.94 | R65.10, R65.11, R65.20, A41.9 |

ICD: International Classification of Diseases.
