# Supplementary material for: Influenza Vaccine Effectiveness in Preventing Influenza A(H3N2)-Related Hospitalizations in Adults Targeted for Vaccination by Type of Vaccine: A Hospital-Based Test-Negative Study, 2011–2012 A(H3N2) Predominant Influenza Season, Valencia, Spain
Source: PLoS One. 2014 Nov 13;9(11):e112294. doi: 10.1371/journal.pone.0112294 (PMC4230985; doi:10.1371/journal.pone.0112294)
Supplement: Table S2 — Influenza vaccination as recorded in the Vaccine Information System for the 2011–2012 influenza season. Vaccine type and healthcare district/hospital. (DOC) [file pone.0112294.s005.doc]

| Table S2. Influenza vaccination by vaccine type and health care district/hospital, as recorded in the Vaccine Information System for the 2011-2012 influenza season. | | | | | | | | | | | |
| --- | --- | --- | --- | --- | --- | --- | --- | --- | --- | --- | --- |
|  | Virosomal* | | Intradermal^a^ | | Classical IM^b^ | | None | | All | |  |
| Health care district/ hospital | n | % | n | % | n | % | n | % | n | % |  |
| General Castellón | 0 | 0 | 132 | 61.7 | 13 | 6.1 | 69 | 32.2 | 214 | 100.0 |  |
| La Plana | 0 | 0 | 88 | 58.3 | 6 | 4.0 | 57 | 37.8 | 151 | 100.0 |  |
| Arnau | 0 | 0 | 90 | 61.2 | 10 | 6.8 | 47 | 32.0 | 147 | 100.0 |  |
| Dr Pesset | 0 | 0 | 156 | 57.8 | 22 | 8.2 | 92 | 34.1 | 270 | 100.0 |  |
| La Fe | 0 | 0 | 71 | 55.0 | 12 | 9.3 | 46 | 35.7 | 129 | 100.0 |  |
| Lluis Alcanyis | 114 | 66.28 | 1 | 0.6 | 10 | 5.8 | 47 | 27.3 | 172 | 100.0 |  |
| San Juan Alicante | 76 | 58.02 | 0 | 0.0 | 19 | 14.5 | 36 | 27.5 | 131 | 100.0 |  |
| Elda | 138 | 60 | 0 | 0.0 | 21 | 9.1 | 71 | 30.9 | 230 | 100.0 |  |
| General Alicante | 83 | 46.37 | 1 | 0.6 | 25 | 14.0 | 70 | 39.1 | 179 | 100.0 |  |
| All | 411 | 25.32 | 539 | 33.2 | 138 | 8.5 | 535 | 33.0 | 1,623 | 100.0 |  |

The Vaccine Information System is a population-based online vaccination registry accessible to public and private sector health care providers in which vaccination date, vaccine, manufacturer, and batch are recorded; this information is linked with a unique personal identification number to clinical and socio-demographic individual records.

IM: intramuscular

^a^ Virosomal and intradermal vaccines were recommended and offered free of charge to inhabitants aged 65 years or older seeking care at the health care facilities located in the health care districts to which a particular vaccine, either virosomal or intradermal, was distributed. Virosomal and intradermal vaccines were distributed by health care district with minor leakage between districts. Only two intradermal vaccines were recorded outside their district of distribution among included subjects.

^b^ The classical intramuscular (IM) influenza vaccine was recommended and offered free of charge to all inhabitants of all health care districts, aged 6 months to under 65 years, belonging to target groups. Among included subjects, 75 (54%) of the recorded 138 classical IM vaccines were administered to subjects aged 65 or older. Those subjects were not included in the by type of vaccine estimates. By type of vaccine estimates were obtained for each vaccine restricted to their distribution area and subjects belonging to the age groups to which the vaccine was offered in the seasonal vaccination campaign.
