# Supplementary material for: Influenza Vaccine Effectiveness in Preventing Influenza A(H3N2)-Related Hospitalizations in Adults Targeted for Vaccination by Type of Vaccine: A Hospital-Based Test-Negative Study, 2011–2012 A(H3N2) Predominant Influenza Season, Valencia, Spain
Source: PLoS One. 2014 Nov 13;9(11):e112294. doi: 10.1371/journal.pone.0112294 (PMC4230985; doi:10.1371/journal.pone.0112294)
Supplement: Table S3 — a. Characteristics of unvaccinated and vaccinated subjects (i); demographic, socioeconomic, smoking habits, body mass index, and number of risk factors. b. Characteristics of unvaccinated and vaccinated subjects (ii): use of healthcare services, previous vaccination, length of stay, intensive care unit admission and in-hospital death. (PDF) [file pone.0112294.s006.pdf]

Supporting Table S3

Puig-Barberà et al., Influenza vaccine effectiveness in preventing influenza A(H3N2)-related hospitalizations in adults targeted for vaccination by type of vaccine: a hospital-based test-negative study, 2011-2012 A(H3N2) predominant influenza season, Valencia, Spain, *PLoS One, 2014*

Correspondence:

Dr. Joan Puig-Barberà,
Vaccines Research Area,
Fundación para el Fomento de la Investigación Sanitaria y Biomédica de la Comunitat Valenciana (FISABIO),
Avenida Cataluña, 21 46020 Valencia, Spain.
Phone: +34 961 925 948 Fax: +34 961 925 938
E-mail: puig_joa@gva.es

Table S3a Characteristics of unvaccinated and vaccinated subjects (i)

		Unvaccinated Number (%) 745 (39)	Vaccinated[a] Number (%) 1,169 (61)	Crude odds ratio (95% CI)	P-value (LR test)
Age, mean in years (s.d.)		71.6 (14.8)	78.3 (10.2)		<0.0001
Age group					
	18-49	73 (84)	14 (16)	1.0	<0.0001
	50-64	138 (55)	112 (45)	4.2 (2.3-7.9)	
	65-74	178 (44)	231 (56)	6.8 (3.7-12.4)	
	75-79	122 (33)	247 (67)	10.6 (5.7-19.5)	
	80-84	106 (31)	235 (69)	11.6 (6.2-21.4)	
	>=85	128 (28)	330 (72)	13.4 (7.3-24.7)	
Sex					
	Male	378 (35)	692 (65)	1.0	0.0003
	Female	367 (43)	477(57)	0.7 (0.6-0.9)	
High-risk conditions					
	None	105 (45)	128 (55)	1.0	<0.0001
	One	301 (45)	373 (55)	1.0 (0.8-1.4)	
	Two or more	339 (34)	668 (66)	1.6 (1.2-2.2)	
Body mass index					
	<18.5	17 (57)	13 (43)	0.5 (.2-1.1)	
	18.5 to 24.9	230 (40)	347 (60)	1.0	0.1684
	25 to 29.9	276 (37)	470 (63)	1.1 (0.9-1.1)	
	30 to 39.9	197 (39)	310 (61)	1.0 (0.8-1.3)	
	>=40	25 (46)	29 (54)	0.8 (0.4-1.3)	
Smoking					
	Never	341 (39)	532 (61)	1.0	<0.0001
	Ex-smoker	249 (31)	548 (69)	1.4 (1.2-1.7)	
	Current smoker	155 (64)	89 (36)	0.4 (0.3-0-5)	
Occupational social class					
	Professional to skilled manual	173 (39)	274 (61)	1.0	0.9127
	Partially skilled- unskilled	572 (39)	895 (61)	1.0 (0.8-1.2)	

CI Confidence Interval
LR Likelihood ratio
a Vaccinated 15 or more days before onset of symptoms.

Table S3b Characteristics of unvaccinated and vaccinated subjects (ii)

	Unvaccinated Number (%) 745 (39)	Vaccinated[a] Number (%) 1,169 (61)	Crude odds ratio (95% CI)	P-value (LR test)
Outpatient visits last three months				
None	204 (48)	220 (52)	1.0	<0.0001
One	164 (41)	236 (59)	1.3 (1.0-1.8)	
Two or more	377 (35)	713 (65)	1.8 (1.4-2.2)	
Times hospitalized previous 12 months				
None	500 (40)	746 (60)	1.0	0.2841
One	162 (38.5)	259 (61.5)	1.1 (0.9-1.3)	
Two	42 (33.3)	84 (66.7)	1.3 (0.9-2.0)	
Three or more	41 /33.9)	80 (66.1)	1.3 (0.9-1.9)	
Symptom onset to swab (days)				
1 to 2	141 (41)	207 (59)	1.0	0.8486
3 to 4	261 (39)	416 (61)	1.1 (0.8-1.4)	
5 to 7	266 (39)	414 (61)	1.1 (0.8-1.4)	
>7	77 (37)	132 (63)	1.2 (0.8-1.7)	
Seasonal 2010 recorded				
No	628 (79)	169 (21)	1.0	<0.0001
Yes	117 (10)	1000 (90)	31.8 (24.6-41.0)	
Seasonal 2009 recorded				
No	582 (76)	188 (24)	1.0	<0.0001
Yes	163 (14)	981 (86)	18.6 (14.8-23.5)	
Pandemic 2009 recorded				
No	662 (49)	679 (51)	1.0	<0.0001
Yes	83 (14)	490 (86)	5.8 (4.5-7.4)	
23 polysaccharide pneumococcal vaccine				
No	666 (46)	793 (54)	1.0	<0.0001
Yes	79 (18)	376 (83)	4.0 (3.1-5.2)	
Length of stay, mean (SD)	7.3 (6.1)	7.2 (6.0)		0.8299
Intensive care unit admission				
No	726 (97)	1.134 (97)	1.0	0.5680
Yes	19 (3)	35 (3)	1.2 (0.7-2.1)	
Died during hospital stay				
No	709 (95)	1,093 (93)	1.0	
Yes	36 (5)	76 (7)	1.4 (0.9-2.1)	0.1310

CI Confidence Interval
LR Likelihood ratio
a Vaccinated 15 or more days before onset of symptoms.
