# Supplementary material for: Influenza Vaccine Effectiveness in Preventing Influenza A(H3N2)-Related Hospitalizations in Adults Targeted for Vaccination by Type of Vaccine: A Hospital-Based Test-Negative Study, 2011–2012 A(H3N2) Predominant Influenza Season, Valencia, Spain
Source: PLoS One. 2014 Nov 13;9(11):e112294. doi: 10.1371/journal.pone.0112294 (PMC4230985; doi:10.1371/journal.pone.0112294)
Supplement: Text S1 — Laboratory procedures. (DOC) [file pone.0112294.s007.doc]

**Text S1. Laboratory procedures**

A nasopharyngeal and a pharyngeal swab were obtained from each included patient. Samples were placed in vials with viral transport medium containing Hank´s Balanced Salt Solution (HBSS), 1M Hepes buffer, 0.5 % gelatin, 7.5% sodium bicarbonate, and antibiotics (penicillin, streptomycin and amphotericin B). All samples were kept at –20ºC until sent to the Instituto Valenciano de Microbiología (Valencian Institute of Microbiology) (IVAMI; http://www.ivami.com) that served as the reference laboratory. Total nucleic acids were extracted from samples using an automatic extraction procedure (Maxwell 16 Viral Total Nucleic Acid Purification kit, Promega), following the instructions of the manufacturer.

Four multiplex real-time RT-PCR / PCR qualitative amplifications were performed in a final volume of 25 microl containing 5.0 microl extracted nucleic acid, 3.75 microl double-distilled water, 12.5 microl TaqMan RT-PCR master mix (2X), 0.6 microl RT-enzyme mix Taqman RNA Ct one Step kit, Applied Biosystems), and 3.15 microl of the primers and probes mixtures, respectively. Primers and probes were selected from previous publications [1-7], but the combinations of reporter and quencher for probes were changed according to our scheme for the combinations in four multiplex real-time amplifications to be read at 520, 550, or 580 nm. All primers and probes mixtures included sense and antisese primers 0.8 M each, and probes 0.2 M each, final concentrations, grouped as follows: multiplex # 1 (influenza virus type AHe 2009 and influenza virus type BSuwannakarn 2008) with the dual labeled probes FAM-BHQ-1 and HEX-BHQ-1, respectively; multiplex # 2 (coronavirus 229-E [7] , coronavirus NL63 [7] , coronavirus HKU1 [7] , metapneumovirus [2] and bocavirus [5]) with dual labeled probes FAM-MGB-NFQ for coronavirus, HEX-BHQ-1 for metapneumovirus, and TAMRA-BHQ2 for bocavirus; multiplex # 3 respiratory syncytial virus (RSV) subgroup A [6] , RSV subgroup B [6], adenovirus [6], parainfluenza virus (PIV) type [6], PIV type [6], PIV type [6] and PIV type [6]) with probes labeled TAMRA-BHQ-2 for both RSV, VIC-MGB-NFQ for adenovirus, FAM-MGB-NFQ for PIV type 1, and FAM-BHQ-1 for PIV types 2, 3 and 4; and multiplex # 4 (rhinovirus [3] and human RNP gene) with probes labeled HEX-BHQ-1 for rhinovirus [3] , and FAM-BHQ-1 for the RNP gene used as a control of the presence of nucleic acid extract and amplification inhibitors. The real-time amplification was done in a OneStep Plus Real Time equipment (Applied Biosystems) and the amplification conditions included 48ºC for 30 minutes, followed by 95ºC for 10 min, and then 45 cycles of 95ºC for 15 seconds and 55ºC for 30 seconds. If positive results were obtained for coronavirus using the primers mixture for types 229-E, NL63 and HKU1 (multiplex 2), RSV using the primers mixture for subgroup A and B or for PIV which used probes with the same quencher and reporter (multiplex 3), a separated amplification with each set of primers separately for each subgroup or type, respectively, was performed. Negative results for viruses were only considered if the human RNP gene amplification was positive. Laboratory procedures to prevent PCR contamination were followed and a series of multiplex assays #1 to #4 negative controls without sample nucleic acid were included in all runs.

**References**

1. He J, Bose ME, Beck ET, Fan J, Tiwari S, et al. (2009) Rapid multiplex reverse transcription-PCR typing of influenza A and B virus, and subtyping of influenza A virus into H1, 2, 3, 5, 7, 9, N1 (human), N1 (animal), N2, and N7, including typing of novel swine origin influenza A (H1N1) virus, during the 2009 outbreak in Milwaukee, Wisconsin. J Clin Microbiol 47: 2772-2778.

2. Matsuzaki Y, Takashita E, Okamoto M, Mizuta K, Itagaki T, et al. (2009) Evaluation of a new rapid antigen test using immunochromatography for detection of human metapneumovirus in comparison with real-time PCR assay. J Clin Microbiol 47: 2981-2984.

3. Tapparel C, Cordey S, Van Belle S, Turin L, Lee WM, et al. (2009) New molecular detection tools adapted to emerging rhinoviruses and enteroviruses. J Clin Microbiol 47: 1742-1749.

4. Suwannakarn K, Payungporn S, Chieochansin T, Samransamruajkit R, Amonsin A, et al. (2008) Typing (A/B) and subtyping (H1/H3/H5) of influenza A viruses by multiplex real-time RT-PCR assays. J Virol Methods 152: 25-31.

5. Neske F, Blessing K, Tollmann F, Schubert J, Rethwilm A, et al. (2007) Real-time PCR for diagnosis of human bocavirus infections and phylogenetic analysis. J Clin Microbiol 45: 2116-2122.

6. van de Pol AC, van Loon AM, Wolfs TF, Jansen NJ, Nijhuis M, et al. (2007) Increased detection of respiratory syncytial virus, influenza viruses, parainfluenza viruses, and adenoviruses with real-time PCR in samples from patients with respiratory symptoms. J Clin Microbiol 45: 2260-2262.

7. Kuypers J, Martin ET, Heugel J, Wright N, Morrow R, Englund JA. (2007) Clinical disease in children associated with newly described coronavirus subtypes. Pediatrics 119: e70-e76.
