# Supplementary material for: Influenza Vaccine Effectiveness in Preventing Influenza A(H3N2)-Related Hospitalizations in Adults Targeted for Vaccination by Type of Vaccine: A Hospital-Based Test-Negative Study, 2011–2012 A(H3N2) Predominant Influenza Season, Valencia, Spain
Source: PLoS One. 2014 Nov 13;9(11):e112294. doi: 10.1371/journal.pone.0112294 (PMC4230985; doi:10.1371/journal.pone.0112294)
Supplement: Text S2 — Bias due to design. (DOC) [file pone.0112294.s008.doc]

**Text S2. Bias due to design**

Bias and confounding due to the test-negative approach could, finally, distort our influenza vaccine effectiveness (IVE) estimates. This could be the case if hospitalization of eligible subjects within seven days of influenza-like-illness (ILI) onset and negative for influenza was dependent on influenza vaccination status, or if the distribution of non-influenza causes of hospitalization did vary by influenza vaccination status [1] Second, the test-negative approach could bias IVE estimates if subjects admitted when influenza was not circulating were included or if calendar time was not taken into consideration [1]. Although IVE estimates obtained by the test-negative approach are described as valid under a wide range of assumptions [1-3] differences in health care-seeking behavior [2], viral interference [2,4], or diverging probability of symptomatic illness by vaccine status [2,5] could bias our estimates.

Our previous published results [6] and a recent paper by Sundaram et al [7] show no association of influenza vaccination with hospitalization due to other respiratory viruses. To assure risk comparability we restricted our overall and subgroup analysis to periods in which influenza was ascertained in the groups studied and in all cases we adjusted for epidemiological week. Regarding the modification of the probability of symptomatic illness by vaccine status it could be argued that in fact this is exactly the desired outcome of vaccination programs. Results that do not support the hypothesis that vaccination mitigates influenza illness severity had been recently published [8].

It is argued that if test-negative design study is used to assess IVE against severe influenza-associated outcomes such as influenza-related hospitalizations, the results of such a study may be of limited relevance if a substantial proportion of patients were hospitalized due to complications of influenza that occur after the virus is no longer detectable [2]. This assertion is supported by a description of a series of nine cases [9]. We have shown, however, that by looking for emergency admissions within seven days of ILI symptoms onset we were able to detect influenza virus presence in a substantial number of admissions that allowed us to report age-specific influenza hospitalization rates [6,10,11] fully consistent with the ones obtained by statistical modeling [12].

**References**

1. Jackson ML, Nelson JC. (2013) The test-negative design for estimating influenza vaccine effectiveness. Vaccine 31: 2165-2168.

2. Foppa IM, Haber M, Ferdinands JM, Shay DK. (2013) The case test-negative design for studies of the effectiveness of seasonal influenza vaccine. Vaccine 31: 3104-3109.

3. De Serres G, Skowronski D M, Ambrose C. (2013) The test-negativee design: validity, accuracy and precision of vaccine efficacy estimates compared to the gold standard of randomised placebo-controlled clinical. Eurosurveillance 18: pii=20585.

4. Cowling BJ, Nishiura H. (2012) Virus interference and estimates of influenza vaccine effectiveness from test-negative studies. Epidemiology 23: 930-931.

5. Ferdinands JM, Shay DK. (2011) Magnitude of Potential Biases in a Simulated Case-Control Study of the Effectiveness of Influenza Vaccination. Clin Infect Dis 54: 25-32.

6. Puig-Barberà J, Díez-Domingo J, Arnedo-Pena A, Ruiz-García M, Pérez-Vilar S, et al. (2012) Effectiveness of the 2010-2011 seasonal influenza vaccine in preventing confirmed influenza hospitalizations in adults: A case-case comparison, case-control study. Vaccine 30: 5714-5720.

7. Sundaram ME, McClure DL, Vanwormer JJ, Friedrich TC, Meece JK, Belongia EA. (2013) Influenza Vaccination is Not Associated with Detection of Non-Influenza Respiratory Viruses in Seasonal Studies of Influenza Vaccine Effectiveness. Clin Infect Dis 57: 789-793.

8. McLean HQ, Meece JK, Belongia EA. (2013) Influenza vaccination and risk of hospitalization among adults with laboratory confirmed influenza illness. Vaccine : [Epub ahead of print].

9. Feldman PS, Cohan MA, Hierholzer WJ. (1972) Fatal Hong Kong influenza: a clinical, microbiological and pathological analysis of nine cases. Yale J Biol Med 45: 49-63.

10. Puig-Barberà J, Díez-Domingo J, García-de-Lomas J, Ruiz-García M, Larrea-González R, et al. Influenza Vaccines for the World. Valencia, Spain, 9-12 October 2012. (Poster 142).

11. Puig-Barberà J, Arnedo-Pena A, Pardo-Serrano F, Tirado-Balaguer MD, Pérez-Vilar S, et al. (2010) Effectiveness of seasonal 2008-2009, 2009-2010 and pandemic vaccines, to prevent influenza hospitalizations during the autumn 2009 influenza pandemic wave in Castellón, Spain. A test-negative, hospital-based, case-control study. Vaccine 28: 7460-7467.

12. Zhou H, Thompson WW, Viboud CG, Ringholz CM, Cheng PY, et al. (2012) Hospitalizations associated with influenza and respiratory syncytial virus in the United States, 1993-2008. Clin Infect Dis 54: 1427-1436.
